# Pharmacogenetic-Based Interactions between Nutraceuticals and Angiogenesis Inhibitors

**DOI:** 10.3390/cells8060522

**Published:** 2019-05-30

**Authors:** Raffaele Di Francia, Massimiliano Berretta, Giulio Benincasa, Alfredo D’Avino, Sergio Facchini, Domenico Costagliola, Paola Rossi

**Affiliations:** 1 Gruppo Oncologico Ricercatori Italiani GORI, 33100 Pordenone, Italy; rdifrancia@libero.it; 2 Department of Medical Oncology, National Cancer Institute, CRO 33018 Aviano, Italy; 3 Pineta Grande Hospital, 81030 Castel Volturno, Caserta, Italy; giulio.benincasa@pinetagrande.it (G.B.); alfredo.davino@pinetagrande.it (A.D.); domenico.costagliola@pinetagrande.it (D.C.); 4 Department of Urology, University Federico II of Napoli, 80126 Naples, Italy; sergio.facchini.93@gmail.com; 5 Department of Biology and Biotechnology “L. Spallanzani”, University of Pavia, 27100 Pavia, Italy; paola.rossi@unipv.it

**Keywords:** neoangiogenesis, complementary and alternative medicines (CAMs), targeted treatment, pharmacogenomics, cytochrome P450 polymorphisms, nutraceuticals, dietary supplements

## Abstract

**Background:** Angiogenesis inhibitors (AIs) have become established as an effective cancer treatment. Whereas their interactions with antineoplastic drugs have extensively been investigated, little is known of the effect of their co-administration with nutraceuticals/dietary supplements (N/DSs), which are often self-prescribed. N/DSs comprise a wide range of products such as herbs, nutrients, vitamins, minerals, and probiotics. Assessment of their interactions with cancer drugs, particularly AIs, is hampered by the difficulty of gauging the amount of active substances patients actually take. Moreover, there is no agreement on which approach should be used to determine which N/DSs are most likely to influence AI treatment efficacy. We present a comprehensive review of the metabolic routes of the major AIs and their possible interactions with N/DSs. **Methods:** The PubMed and Cochrane databases were searched for papers describing the metabolic routes of the main AIs and N/DSs. **Results:** Data from the 133 studies thus identified were used to compile a diagnostic table reporting known and expected AI-N/DS interactions based on their metabolization pathways. AIs and N/DSs sharing the cytochrome P450 pathway are at risk of negative interactions. **Conclusions:** Recent advances in pharmacogenetics offer exceptional opportunities to identify prognostic and predictive markers to enhance the efficacy of individualized AI treatments. The table provides a guide to genotyping patients who are due to receive AIs and is a promising tool to prevent occult AI-N/DS interactions in poor metabolizers. N/DS use by cancer patients receiving AIs is a topical problem requiring urgent attention from the scientific community.

## 1. Introduction

The growth of bulky tumors and metastatic masses depends on the formation of new blood vessels, i.e., angiogenesis [1]. The angiogenesis process consists of a sequence of multiple steps that are regulated by endothelial cells (ECs), which receive an angiogenic stimulus. EC migration is accompanied by their proliferation and organization into three-dimensional structures that invade perivascular structures. EC production may result not only from the division of existing differentiated ECs, but also from the influx of bone marrow-derived circulating endothelial progenitor cells, a process that is called systemic vasculogenesis [2]. Angiogenesis can occur either by sprouting (branching of new capillaries from existing vessels) and non-sprouting processes (EC multiplication in vessel walls) [3]. The latter mechanism is closely dependent on vascular endothelial growth factor (VEGF), a vascular permeability factor [4,5,6]. 

VEGF is a homodimeric heparin-binding glycoprotein of which at least four isoforms—VEGF_121_, VEGF_165_, VEGF_189_, and VEGF_205_—have been described based on the number of amino acids contained in each protein as obtained by alternative mRNA splicing. All these isoforms are referred to as VEGF-A, which is involved in non-sprouting angiogenesis. VEGF-B seems to play an important role in vasculogenesis but may also have other functions such as activation of enzymes that enhance EC multiplication [6,7]. VEGF-C is most commonly associated with lymphangiogenesis. The role of VEGF-D is less clear, but it seems to bind to VEGF receptors (VEGFRs)-2 and -3 and to induce angiogenesis in vivo. These VEGF variants exert their angiogenic activity through specific receptors. The VEGFRs that are most closely involved in angiogenesis are VEGFR-1 (FLT-1) and VEGFR-2 (KDR/Flk-1). VEGFR-1 and -2 are expressed in certain types of vascular ECs and tumor cells. VEGFRs are members of the receptor tyrosine kinase (TK) family, which comprises seven extracellular immunoglobulin-like domains containing the TK domain. VEGF binding to its receptor causes VEGFR dimerization and, through it, activation of intrinsic TK [8]. Despite the high homology of the TK domain, different VEGFRs are characterized by different signals. VEGFR-1 plays a regulatory role in angiogenesis and is weakly autophosphorylated by VEGF in ECs. Besides receptor TK pathways, signaling activated by cell-cell or cell-matrix interactions—especially the downstream RAS/RAF/MEK/ERK pathway and activated Rac1—also has a key role [9]. 

Current antiangiogenic treatments are mainly based on monoclonal antibodies (mAbs) and small molecules with TK inhibitor (TKI) activity [10]. In the past 15 years these agents have radically changed the therapeutic protocols for several tumors, including liver, lung, breast, pancreatic and colorectal cancer [11,12,13,14], not least because targeted drugs are better tolerated than conventional chemotherapy [15,16]. 

Nutraceuticals and dietary supplements (N/DSs) comprise a wide range of products that include herbs, nutrients, vitamins, minerals, and probiotics. Cancer patients are known to self-prescribe natural remedies [17]. It is well known the anti-tumor activity of these biomolecules when studied as single agents. Thus, the mechanism of action and the metabolic route is still unclear when present in whole food. Furthermore, it is hypothesized that the mixture of these molecules present in food have more anticancer effects rather than when administered as single isolated agent [18]. 

Targeted cancer therapies are mostly based on monoclonal antibodies or TKIs. Monoclonal antibodies are metabolized by esterase and share no signaling pathways with N/DSs. In contrast, TKIs are small molecule inhibitors (SMIs) that are preferentially metabolized by cytochrome P450 (CYP450), a pathway that they share with N/DSs. Notably, agents that target the VEGF ligand-receptor system have shown relatively little toxicity compared to standard chemotherapeutics in clinical trials.

Although the concomitant use of angiogenesis inhibitors (AIs) and N/DSs is quite common, data on the effects of their co-administration are still limited. Notably, different AI-N/DS interactions are seen in different patients, due to factors such as polypharmacy, age, gender, comorbidities and variability in the genes that encode drug-metabolizing enzymes or drug transporters. Although AI-N/DS co-administration is not necessarily contraindicated, it should be monitored and adjusted for dosage. Yet, little is known about the interactions between N/DSs and AIs, including their magnitude and clinical relevance. Indeed, very few studies have addressed the topic, and our knowledge is largely based on the report of clinical cases. Since the CYP450 family plays a central role in the metabolic routes of many AIs, co-administration of VEGFR TKIs and CYP450 inhibitors/inducers should be avoided [19].

This work reviews the current Food and Drug Administration (FDA)-approved AIs and their interactions with the most common N/DSs. These data are offered as a guide to help patient evaluation based on their genetic and metabolic profile. They are not intended to replace patient-tailored treatment; rather, they provide an additional source of information that can be used in counseling about N/DSs, treatment monitoring, and dose adjustment.

## 2. Materials and Methods

To establish the current knowledge of AI-N/DS interactions, the PubMed and Cochrane databases and the grey literature were searched for relevant papers using the keywords “angiogenesis inhibitors,” AND “drug interactions.” In the second step “metabolism”, “nutrients”, “herbal”, “Complementary and Alternative Medicines” (CAMs), and “vitamins” were added singly. In the third step, the search was further narrowed using the terms “pharmacogenetics” and “TKI metabolism”. There were no publication date constraints; the search was limited to the English literature. The 2437 papers retrieved by the search were examined by two authors (RDF and MB).

Non-relevant papers, papers that did not mention AIs, duplicates, and works with equivocal titles were excluded. Of the studies conducted by the same institution at different times, the most recent and complete was included unless they involved different methods/endpoints or focused on specific issues. Papers whose full text or at least the abstract were not available were also excluded. The reference lists of the papers that were included were searched for any other relevant articles.

Publication bias was assessed using Egger’s tests [20], whose results showed that there was no publication bias (*p* = 0.354 for AIs and *p* = 0.421 for pharmacogenetic interactions).

## 3. Results

Data from 22 of the 2437 studies thus identified were used to compile a diagnostic table reporting known and expected AI-N/DS interactions based on their metabolization pathways. AIs and N/DSs sharing the cytochrome P450 pathway are at risk of negative interactions. 

The hits of the second step were 52 papers that had passed the abstract screening approach: “metabolism”, 25 papers; “nutrients”, no paper; “vitamins”, 42 papers; “Complementary and Alternative Medicines”, 15 papers; “vitamins”, 42 papers. In parallel, “pharmacogenetics” and “TKI metabolism” allowed retrieving 147 and 189 paper, respectively, of which only 51 were relevant and not redundant. 

**Figure 1:**
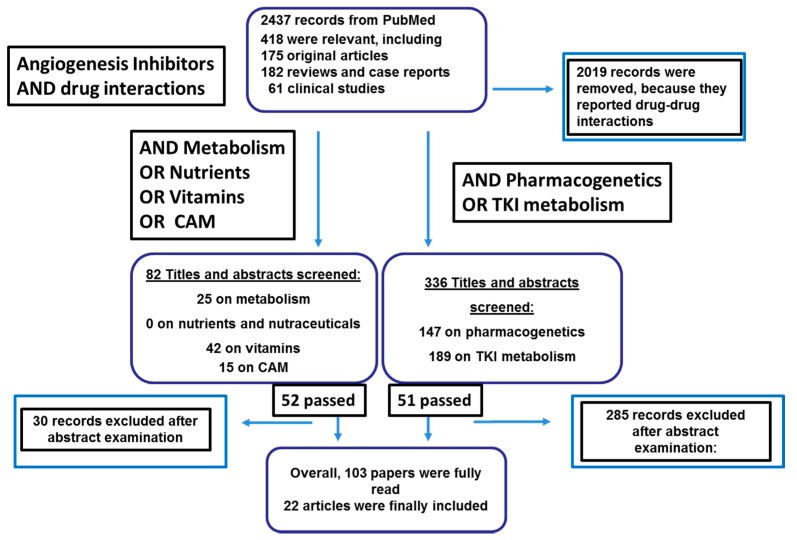
Literature research about angiogenesis inhibitors’ (AIs) and nutraceuticals’ interactions.

Overall, 103 studies addressing drug metabolism primarily in relation to CYP450 polymorphisms were reviewed by reading them fully and 22 were included in the reference list. The study retrieval and selection process is outlined in Figure 1. Some papers were excluded because they were redundant (*n* = 2019), they did not report pharmacogenetic results (*n* = 81), they were in vitro studies (*n* = 74), studies of animal models (*n* = 38), or uninformative reviews or comments (*n* = 29).

## 4. Pathophysiology of Angiogenesis

Angiogenesis is the process by which new blood vessels are created from the existing vasculature. It drives physiological processes such as organ growth, repair, and functioning, placenta formation, embryo development, and tissue remodeling, regeneration, and engineering [21]. The growth process targets vascular ECs and is driven by stimuli from specific angiogenic factors that activate complex molecular mechanisms [22,23].

Abnormal angiogenesis is involved in the pathogenesis of a number of diseases including some cancers. Therefore, research into the mechanisms underlying new vessel formation has the potential to inspire new therapeutic strategies as well as new methods to evaluate angiogenesis.

The angiogenesis process consists of three successive stages. 

The first stage involves selection inside capillaries of special ECs called “tip cells”, which initiate the angiogenic expansion. Tip cells play a crucial role in EC invasion and migration and react to stimulation by VEGF-A and VEGFR [24]. These interactions lead tip cells to express Notch family receptors and their transmembrane ligand DLL4 (Delta-like ligand 4) [25,26]. In this way, tip cells guide the VEGF gradient, starting the sprouting process in the new vessel.

The second stage, involving EC migration and proliferation and tube formation, is also mediated by interactions with VEGF-A and VEGFR [27]. It is unclear how the morphogenic events, like tubular sprouting, fusion, and network formation are regulated. However, tip cell migration depends on VEGF-A and their proliferation is regulated by VEGF-A concentration. 

In the third stage, maturation of newly formed vessels involves inhibition of EC proliferation and migration of newly formed capillaries. Subsequently, stabilization involves fusion of the new vessels with other existing vessels to form a tube or a loop. Mural cells such as pericytes and vascular smooth muscle cells exert a key role; in particular, pericytes are in direct communication with ECs and are incorporated into new capillaries to form and stabilize them [28,29]. This mechanism is mainly mediated through platelet-derived growth factor (PDGF)-B and its receptor PDGFR-B [30]. Like VEGFRs, PDGFRs are transmembrane proteins with an intracellular domain exerting TK activity [31].

Other growth factors besides VEGF contribute to vessel development and promote angiogenesis. They include fibroblast growth factor (FGF), transforming growth factor, PDGF, angiopoietins, and receptor TEK tyrosine kinase, endothelial 2 (TIE-2), which are expressed in vascular and stromal cells. When the trigger of the angiogenic process is inflammation, other mediators such as cytokines, prostaglandins, and nitric oxide enhance the angiogenic activity of growth factors [31,32,33,34,35].

### Vascular Endothelial Growth Factor (VEGF) Polymorphisms

Several relationships have been described between VEGF-A polymorphisms and AI treatment outcome, albeit with conflicting results (Table 1).

A trial involving two arms has found that the AA genotype of the VEGF polymorphism −2578C > A (rs699947) and −1154G > A (rs1570360) in the 5′untranslated promoter region (5′UTR) was related with higher overall survival (OS) in patients receiving combined paclitaxel/bevacizumab [36]. 

Single nucleotide polymorphisms (SNPs) of the genes involved in the angiogenesis pathway have been investigated to assess disease risk [39]. A large-scale study has identified four SNPs in the 5′UTR of VEGF-A that were associated with a higher risk of bladder cancer: i) the TT genotype of Ex1-73C > T (rs25648); ii) the AA genotype of −15648A > C (rs833052); iii) the TT genotype of −9228G > T (rs1109324); and iv) the TT genotype of −8339A > T (rs1547651). In addition, another intronic SNP, VEGF-A IVS2 + 1378C > T (rs3024994), was associated with reduced bladder cancer risk (CT genotype) [37]. Moreover, the VEGF-A A allele −2578C > A (rs1547651) and C allele −1498T > C (rs833061) have been associated with a heightened risk of breast cancer (BC) [38]. VEGF-A alleles −460C (rs833061) and +405G (rs2010963) have been related to reduced OS after BC diagnosis, and the combination haplotype −460T/+405C/+936C (rs3025039) has been reported to be significantly associated with increased OS [40]. A lower risk of bone metastasis in BC patients has been related to the CC genotype of VEGF-A − 1498T > C (rs833061) [41]. In contrast, another study has found no association between VEGF-A variants and the risk of developing BC [42], colorectal cancer [43], prostate cancer [44], and non-small cell lung cancer [45].

Also, selected variants in other VEGF pathway candidate genes, including FLT-1, KDR and Nitric Oxide Synthase (NOS3), were not associated with BC risk [38]. These authors reported that the NOS3 − 786TT (rs2070744) and 894GG (rs1799983) genotype were associated with a greater probability of developing metastatic BC. Further work is clearly needed to clarify the role of the gene variants involved in the angiogenesis pathway in disease etiology and treatment outcomes.

## 5. Current Angiogenesis Inhibitors

Recently, the identification of cellular signaling pathways playing a key role in tumor angiogenesis has led to the development of new molecules for targeted therapies [10]. The data regarding AI metabolic pathway and their interactions with N/DSs summarized in Table 2.

### 5.1. Bevacizumab 

Bevacizumab, the first AI to be developed and the first agent to be approved in combination with conventional antineoplastic therapy, was an anti-VEGF monoclonal antibody. By deactivating this ligand, bevacizumab reduces angiogenesis by blocking VEGFR activation. It has achieved encouraging effects both alone and in combination with several antibiotics (e.g., Gemox, Folfox) or TKIs. The main adverse event associated with its administration is bleeding [47]. At present no clinical trials are planned to investigate the effects of co-administration with N/DSs.

### 5.2. Brivanib 

Brivanib is a TKI with a high VEGFR and FGF receptor (FGFR) binding ability. It exerts antitumor activity in sorafenib-resistant patients [48]. BRISK, a large randomized phase III study involving patients at risk of hepatocellular carcinoma (HCC), provided unsatisfactory results [49]. The BRISK-PS (brivanib post-sorafenib) trial, which assessed brivanib vs. placebo in patients with disease progression or intolerance to sorafenib (NCT00825955), failed to meet the primary endpoint of a significant OS improvement (9.4 months vs. 8.2 months, *p* = 0.3307) [49], and also demonstrated the non-inferiority of brivanib to sorafenib [50]. No clinical trials are investigating its interactions with N/DSs.

### 5.3. Cabozantinib

Cabozantinib S-malate is the s-malate salt form of cabozantinib, an orally bioavailable TKI with potent antineoplastic activity. It strongly binds to and inhibits several TK receptors such as VEGFR-1–3. In addition, it induces simultaneous inhibition of hepatocyte growth factor receptor (cMET) and RET, which is often overexpressed in a variety of cancer cell types. Finally, it induces tumor regression because it is also active against FMS-like tyrosine kinase 3 (FLT-3), mast/stem cell growth factor (cKIT), TIE-2, and tropomyosin-related kinase B (TRKB) [51]. In an experimental metastatic mouse model, cabozantinib reduced the number of lung and liver metastases. VEGFR-2 and MET blocking by cabozantinib has significant antitumor activity in HCC and renal cancer and cMET overexpression may be a promising biomarker of efficacy [52]. Pharmacokonetic interaction was found with food. Cobazatinib must be delivered one hour before eating and/or 2 h post-eating [53].

### 5.4. Cediranib 

Cediranib (AZD2171) is a multitargeted inhibitor of VEGFR, KIT, PDGFR-β, and FLT-4. Its potential to interact with CYP450 enzymes is held to be low because cediranib is not a substrate, inhibitor, or inducer of CYP450 enzymes at systemic concentrations. Cediranib has failed to inhibit uridine 5’-diphospho-glucuronosyltransferase (UGT) enzymes at clinically relevant concentrations. However, potential drug–drug interactions involving flavin monooxygenase (FMO) 1 and 3 have not been explored. The H2 antagonists, cimetidine and ranitidine, are substrates for FMO3 and have been used to phenotype FMO3 in vivo [54]. Cimetidine or ranitidine should be administered before paclitaxel, because they interact with the pharmacokinetics of cediranib.

The potential of cediranib to interact with transporters has been investigated for P-glycoprotein (Pgp), breast cancer resistance protein (BCRP), and the hepatic uptake transporters OATP1B1 and OATP1B3. Cediranib is a substrate for P-glycoprotein but not for BCRP, OATP1B1, OATP1B3. In an in vivo interaction study, ketoconazole was found to increase cediranib exposure by 21%. Since cediranib is not a substrate for CYP450 enzymes, the increase can be assumed to be due to inhibition of intestinal absorption. Because of the limited effect of ketoconazole, dosage recommendations are not required when cediranib is co-administered with P-glycoprotein inhibitors. Cediranib has the potential to inhibit Pgp, BCRP, OATP1B1, OATP1B3, and solute carrier family 47 member 1 (SLC47A1 alias MATE1) at higher than systemic concentrations and is a potent inhibitor of MATE2K and OATP1A2 at clinically relevant concentrations [54]. Cediranib must be delivered one hour before eating and/or 2 h post-eating, due for pharmacokinetics interactions.

### 5.5. Everolimus 

The phosphatidylinositol 3-kinase(P3K)/AKT pathway is the mammalian target of rapamycin (mTOR) pathway, a key regulator of growth, angiogenesis, and tumor cell survival. Since it is involved in the development of multiple cancers, mTOR inhibition with everolimus has been investigated in combination with other drugs in several randomized trials [55]. Works investigating the PI3K/AKT/mTOR signaling cascade have described the synergy between everolimus and sorafenib used as HCC suppression therapy. AI activity occurs at separate stages along two pathways, and their combination should be more effective and complementary. Important interactions have been found between everolimus and grape juice [56]. 

### 5.6. Lenvatinib 

Lenvatinib is a TKI that selectively targets a number of molecules like the FGFR-1-4, VEGFR-1-3 (FLT-4), PDGFR, RET, and KIT signaling networks. In a recent trial it significantly improved progression-free survival in patients with differentiated thyroid cancer refractory to radioiodine (iodine-131) therapy [57].

The main metabolic pathways in humans are oxidation by aldehyde oxidase, demethylation via CYP3A4, glutathione conjugation with elimination of an O-aryl group (chlorophenyl moiety), and combinations of these pathways followed by further biotransformation (e.g., glucuronidation, hydrolysis of the glutathione moiety), N/DSs have been found to exert no significant influence on lenvatinib administration. Furthermore, since exposure to lenvatinib is amplified in patients with severe hepatic injury, a dose reduction needs to be considered in these individuals [58]. 

### 5.7. Linfatinib

Linifanib (ABT-869) is an orally administered selective VEGFR and PDGFR inhibitor. Administered alone it showed good antitumor activity (OS: 9.7 months; time to progression: 3.7 months) [59]. No data are available about its interactions with N/DSs.

### 5.8. Nintedanib

Nintedanib (BIBF 1120) is a recently developed orally available TKI of VEGFR 1–3, FGFR, and PDGFR. It has been reported to inhibit tumor growth and angiogenesis in a xenograft model and to exhibit moderate effects on ex vivo HCC cell lines [60].

Its predominant metabolic activity is hydrolytic cleavage by esterase, resulting in the free acid moiety BIBF 1202. BIBF 1202 is subsequently glucuronidated by UGT enzymes (UGT 1A1, 1A7, 1A8, and 1A10) to BIBF 1202 glucuronide. The biotransformation of nintedanib affects CYP pathways in a very limited manner, CYP3A4 being the enzyme most closely involved. The main CYP-dependent metabolite has not been detected in plasma in the human ADME study [60]. In vitro, CYP450-dependent metabolism accounted for about 5% compared to about 25% of ester cleavage. Since nintedanib, BIBF 1202, and BIBF 1202 glucuronide have not inhibited or induced CYP enzyme activity in preclinical studies, drug-drug interactions with CYP substrates, inhibitors, or inducers are not expected [61].

### 5.9. Ramucirumab

Ramucirumab is a wholly humanized mAb designed to bind selectively the extracellular domain of VEGFR-2. As a result, it inhibits ligand-stimulated activation of VEGFR-2 and its downstream signaling components, including p44/p42 mitogen-activated protein kinase (MAPK), thus neutralizing ligand-induced proliferation and migration of human ECs [62]. The reason why it is more active in these circumstances is still unclear; ramucirumab will therefore be further developed in a population with elevated alpha-fetoprotein (AFP) after sorafenib failure (progression or intolerance) [63]. No interactions with N/DS have been annotated to date.

### 5.10. Regorafenib

Regorafenib is a novel diphenylurea multikinase inhibitor of VEGFR1-3, c-KIT, TK with immunoglobulin-like and EGF-like domains, PDGFR-2, FGFR-1, RET, BRAF, and p38 MAPK [64]. Although it is structurally related to sorafenib, the addition in the phenyl ring b of a fluorine atom may enhance efficacy. It has also been used to treat metastatic gastrointestinal stromal tumors after the failure of imatinib and sunitinib [65].

Regorafenib is metabolized primarily in the liver by CYP3A4-mediated oxidative metabolism and UGT1A9-mediated glucuronidation. Two major and six minor metabolites have been identified in plasma. The main circulating metabolites in human plasma are M-2 (*N*-oxide) and M-5 (*N*-oxide and *N*-desmethyl), which are pharmacologically active and have similar concentrations as regorafenib in the steady state. M-2 is further metabolized by CYP3A4-mediated oxidative metabolism and UGT1A9-mediated glucuronidation. Metabolite reduction or hydrolyzation by the microbial flora in the gastrointestinal tract allows resorption of the unconjugated active substance and of metabolites (enterohepatic circulation). Furthermore, the components of ginger extract have been reported to possess a favorable pharmacokinetic profile related to interactions with CYP2D6 [66].

### 5.11. Sorafenib 

Sorafenib is an oral TKI that acts on tumor cell proliferation by targeting RAF/MEK/ERK signaling at the level of RAF kinase. Its antiangiogenic effect is produced by blockage of VEGFR-2/-3 and PDGFR-2 TK activity [67]. Sorafenib is metabolized almost exclusively by CYP3A4 and CYP3A5. Caution is required with co-administration of several CYP3A4 inhibitors and other substances, primarily herbal remedies like black cohosh, cascara sagrada, senna, and St John’s wort.

The black cohosh (*Cimicifuga racemosa*) rhizome has been suspected in rare cases of liver injury ranging from abnormal liver function tests and jaundice to various forms of hepatitis and liver failure (often requiring transplantation) in CYP3A4 poor metabolizers (PMs) [68]. Onset was usually within the first three months of regimen initiation. Several patients treated with sorafenib experienced toxicity after taking complementary herbal medicines like black cohosh. Unfortunately, the adverse events were seldom well documented, both due to the absence of data on the specific herbal formulation and dose used and because of multiple confounding factors. In 2006, a review of 42 such cases by the European Medicines Agency (EMEA) and the Committee on Herbal Medicinal Products (HMPC) resulted in the release of a statement indicating a potential connection between products containing *C. racemosa* rhizome and human hepatotoxicity. After reviewing data from more than 40 cases, received through their reporting system and similar systems in other countries and the literature, the UK Medicines and Healthcare products Regulatory Agency (MHRA) also issued an assessment report supporting a causal association. 

Overuse of certain laxatives, like cascara sagrada and senna, can result in electrolyte loss and increase the risk of arrhythmia in patients treated with drugs that prolong the QT interval. Electrolyte disturbances including hypokalemia and hypomagnesemia have been reported with laxative abuse during treatment with sorafenib.

St John’s worth is a potent CYP3A4 inducer. Co-administration with other potent CYP3A4 inducers may reduce the plasma concentrations of sorafenib, which is partially metabolized by the isoenzyme. Administration of a single oral dose (400 mg) of sorafenib to healthy PM volunteers after treatment with a potent CYP3A4 inducer involved a 37% reduction of the mean systemic bioavailability of the drug compared to sorafenib administered alone [19]. 

Until additional information becomes available, patients treated with sorafenib and other potentially hepatotoxic substances metabolized by CYP3A4 should avoid black cohosh. Such substances include acetaminophen; alcohol; androgens and anabolic steroids; antituberculous agents; azole antifungals; Angiotensin Converting Enzyme (ACE) inhibitors; cyclosporine (high dosages); disulfiram; endothelin receptor antagonists; interferons; ketolides and macrolides; kinase inhibitors; minocycline; non-steroidal anti-inflammatories; nucleoside reverse transcriptase inhibitors; proteasome inhibitors; retinoids; sulfonamides; tamoxifen; thiazolidinediones; tolvaptan; vincristine; zileuton; anticonvulsants like carbamazepine, hydantoins, felbamate, and valproic acid; lipid-lowering medications such as fenofibrate, lomitapide, mipomersen, niacin, and statins; and other herbal remedies and N/DSs such as chaparral, comfrey, kava, pennyroyal oil, and red yeast rice [69].

### 5.12. Sunitinib

The label information recommends avoiding co-administration with St. John’s wort (a CYP3A4 inducer) and grapefruit juice (a CYP3A4 inhibitor). A recent work has shown that grapefruit juice increases the area under the concentration-time curve of sunitinib by 11% which, given the significant interindividual variability in drug absorption, is clinically irrelevant [70]. These data can be explained by the fact that grapefruit juice inhibits intestinal CYP3A4 but not hepatic CYP3A4 [71].

### 5.13. Trebananib

Angiopoietins are vascular growth factors that bind to TIE-2 receptor. Angiopoietin-1 mediates vessel maturation, adhesion, migration, and survival, whereas Ang-2 promotes cell death and disrupts vascularization. Trebananib (AMG386) is an AI compound that sequesters Angiopoietin-1 and -2, preventing their interaction with TIE-2 receptor [72].

### 5.14. Vatalanib

Vatalanib (PTK787), a TKI that binds directly to the VEGFR ATP-binding sites, inhibits FLT-1, Flk-1/KDR, and other class III receptor TKs such as PDGFR-2, FLT-4, c-KIT and c-FMS [73]. In vitro experiments with human liver microsomes and hepatocytes have indicated that vatalanib is a substrate of human CYP3A4 and CYP2D6, and partly of CYP1A2. Vatalanib is predominantly metabolized by CYP3A4, which accounts for approximately 95% of cytochrome P450-dependent metabolism [74].

## 6. Conclusions and Future Direction

Angiogenesis is a dynamic physiological process that is mainly induced by tissue hypoxia or ischemia. In neoplastic tissue, it plays a key role in tumor growth and metastatic diffusion. Exploration of the biology of angiogenesis has led to the identification of new prognostic factors, tumor markers, and therapeutic approaches [75]. Prognostic biomarkers of angiogenesis (i.e., VEGF, SNPs) have the potential to provide an important contribution to AI therapies [76].

The benefits of combining N/DSs with AIs, antiblastic chemotherapy, or other targeted agents are being tested in preclinical and clinical trials of several malignancies [77]. However, choosing the most effective combinations requires a better knowledge of the mechanisms by which the antiangiogenetic effects are achieved or the new strategies reach their molecular targets [78]. Recent advances provide exceptional opportunities to identify the genetic profile of those patients who will benefit from targeted therapy and to exclude those who are at high risk of severe toxicity [79,80]. The efficacy of antiangiogenic treatments could be enhanced by incorporating validated N/DSs into treatment regimens, especially where “frail” patients are concerned [81,82]. However, data regarding AI-N/DS interactions are still too limited. Most of the evidence comes from reports of single cases, of studies of animal model, or of in vitro investigations. However, it is difficult to translate this evidence into clinical practice. Yet, the problems hampering the management of drug–N/DS interactions should not be a reason to ignore them, since they may explain some treatment failures.

In conclusion, a better understanding of the causes of the high interindividual variability of drug–N/DS interactions requires large clinical trials. CYP450 genotyping is expected to lead to the development of personalized treatment regimens based on synergistic combinations of AIs and N/DSs [83,84]. Last but not least, a multidisciplinary approach involving oncologists, geneticists, dieticians, and health professionals with advanced pharmacogenomic skills would improve the planning, delivery, and outcome of patient-tailored interventions while optimizing costs and benefits. We think that to reach this challenge of a targeted intervention we need a multidisciplinary approach.

## Figures and Tables

**Table 1:** Known vascular endothelial growth factor (VEGF) polymorphisms and genes associated with VEGF pathways.

Gene cDNA Position	rs	Study Annotation	Ref
**VEGF − 2578C > A**	rs699947	The AA genotype is associated with higher overall survival in patients receiving combined paclitaxel/bevacizumab	[36]
**VEGF − 1154G > A**	rs1570360		
**VEGF-A Ex1 − 73C > T**	rs25648	The TT, AA, TT, and TT genotypes are associated with an increased risk of bladder cancer	[37]
**VEGF-A − 15648A > C**	rs833052		
**VEGF-A − 9228G > T**	rs1109324		
**VEGF-A − 8339A > T**	rs1547651		
**VEGF-A IVS2 + 1378C > T**	rs3024994	Associated with reduced bladder cancer risk	[37]
**NOS3 − 786TT**	rs2070744	Associated with metastatic breast cancer	[38]
**NOS3 894GG**	rs1799983		[38]

**Table 2:** Main characteristics of the major angiogenesis inhibitors and possible pharmacokinetics interactions ^$^ [46].

Drug (alias)	Targets (Type)	Metabolic Route	Possible N/DS Interactions	Pharmacogenomics-Based Warnings
**Bevacizumab**	VEGF (mAb)	Serum esterase	ND	ND
**Brivanib**	VEGFR, FGF (TKIs)		ND	ND
**Cabozantinib**	c-MET, RET, VEGFR1-3, c-KIT (TKIs)	CYP3A4 CYP2C8	Food and N/SD alter PK	Acquired mutation on FLT-3 ITD, c-Kit D816V
**Cediranib**	VEGFR-2, PDGFR, c-KIT (TKIs)	UGTA1A, FMO1, FMO3	Food and N/SD alter PK	ND
**Everolimus**	m-TOR	CYP2C19 CYP3A4	Grapefruit juice can increase the blood levels and effects of everolimus	Screening for SNPs CYP2C19*2, *3 and *17
**Lenvatinib**	VEGFR1-3, FGFR-1, PDGFRα, RET, KIT (TKIs)		ND	acquired VEGFR-2 mutation
**Linfantib**	VEGFR-2, PDGFRs (TKIs)	ND	ND	ND
**Nintedanib**	VEGFR-1-3, FGFR-1, PDGFR (TKIs)	ND	ND	ND
**Ramucirumab**	Selective VEGFR-2 mAb	Serum esterase	ND	ND
**Refametinib**	MEK 1-2, MAPK inhibitor	ND	ND	ND
**Regorafenib**	VEGFR1-3, c-KIT, TK Ig-like, EGF-like PDGF-2, FGF-1, RET, BRAF, MAPK (TKIs)	ND	ginger	ND
**Sorafenib**	VEGFR-2, PDGFR, c-KIT, BRAF (TKIs)	CYP3A4, CYP3A5	black cohosh, cascara sagrada, senna, St. John’s wort	ND
**Sunitinib**		CYP3A4	St. John’s wort	Dose reductions for CYP3A4 Poor Metabolizers
**Trebananib**	TIE-2 (Ang) AI		ND	ND
**Vatalanib**	VEGFR-1,-2,-3, PDGFRs, c-FMS (TKIs)	CYP3A4	ND	ND

^$^ Source: https://www.drugbank.ca/drugs/DB11958. Abbreviation: Cytochrome P450 family (CYP); Receptors for growth factors (VEGFR, FGFR, PDGFR); tyrosine kinases (TKs) and the downstream RAS/RAF/mitogen-activated protein extracellular kinase (MEK)/extracellular signal-regulated kinase (ERK). Pharmacokinetic (PK); not described (ND).
